# Quick communication: Active surveillance of rat sarcoma viral oncogene homolog-positive indeterminate thyroid nodules with categories III and IV, the Bethesda System for Reporting Thyroid Cytopathology, in thyroidology for thyroidologists

**DOI:** 10.1590/1806-9282.20260147

**Published:** 2026-09-28

**Authors:** Ilker Sengul, Demet Sengul

**Affiliations:** 1 Giresun University, Faculty of Medicine, Thyroidology Unit – Giresun, Turkey.; 2 Giresun University, Faculty of Medicine, Division of Endocrine Surgery – Giresun, Turkey; 3 Giresun University, Faculty of Medicine, Department of General Surgery – Giresun, Turkey.; 4 Giresun University, Faculty of Medicine, Department of Pathology – Giresun, Turkey.

Indeterminate cytology remains a major source of controversy in the management of suspicious thyroid nodules in thyroidology today^1-10^. The prospective analysis by Wu et al.^1^, offers a timely provocation regarding the clinical management of RAS-mutated indeterminate^1-7^ thyroid nodules, just the Categories III and IV, the Bethesda System for Reporting Thyroid Cytopathology (TBSRTC), in thyroidology^1-10^. By demonstrating that immediate resection may lead to overtreatment in up to 50% of cases, the authors provide a necessary evidentiary basis for active surveillance (AS) in thyroidology. However, a multifaceted interrogation of their data—particularly the longitudinal growth dynamics and the nuanced molecular landscape—suggests that our understanding of "Rat sarcoma viral oncogene homolog (RAS)-positivity" requires a more granular, isoform-specific refinement. The most striking, yet perhaps under-scrutinized, finding in Wu et al.^1^ cohort is the nonlinear growth trajectory of these nodules. While volume remained remarkably stable during the first 24 months (2% cumulative growth), a sharp escalation to 24% growth was observed by the 4-year mark. This "growth spurt" challenges the assumption of perpetual indolence and suggests a biological "tipping point." One might ask: Is this delayed progression indicative of a late-stage clonal expansion or a manifestation of specific RAS isoforms? As such, evidence increasingly suggests that the clinical "aggressiveness" of a RAS mutation is not monolithic but isoform-dependent. In addition, while Neuroblastoma-rat sarcoma viral oncogene homolog (N-RAS) mutations often correlate with a more indolent, Non-invasive Follicular Thyroid Neoplasm with Papillary-like Nuclear Features (NIFTP)-like phenotype, Harvey-rat sarcoma viral oncogene homolog (H-RAS) and Kirsten-rat sarcoma viral oncogene homolog (K-RAS) isoforms have been associated with a higher propensity for structural progression and, in some contexts, higher-grade histopathology. Furthermore, the observation that nuclear atypia was significantly more prevalent in the surgical group (52 vs. 38%) reinforces the necessity of integrating cytomorphological nuances with molecular data. This is particularly relevant in light of the 2023 TBSRTC, 3rd edition, which emphasizes the subcategorization of Atypia of undetermined significance (AUS) to better predict malignancy risks^2-5^. Moreover, the transition of four patients to surgery—resulting in low-risk malignant histology—may validate the safety of deferred intervention but simultaneously underscores the limitations of binary molecular testing. Herein, without accounting for secondary co-mutations or specific RAS isoforms, we risk applying a "one-size-fits-all" surveillance protocol to a biologically heterogeneous group of lesions. We might liken this situation to an observer who, upon receiving a storm warning (RAS mutation), decides whether to stay outside by examining the structure of the clouds in the sky and the wind speed (clinical factors and ultrasound) instead of immediately taking shelter. Wu et al.^1^ attempted to show us with evidence that not every cloud brings a storm. Yet, the 4-year data might serve as a reminder that the horizon remains unpredictable. Intercalary, the authors evaluated indeterminate cytology^1-7^ solely by including Categories III and IV, excluding Category V. Future management frameworks must transcend the "positive/negative" dichotomy, moving toward a multi-parametric risk score that integrates isoform specificity, volumetric doubling time, and the refined TBSRTC criteria from the last edition^2-5^. Only then can we truly fulfill the promise of personalized thyroidology for thyroidologists. *Felix qui potuit rerum cognoscere causas*.

## Data Availability

The datasets generated and/or analyzed during the current study are available from the corresponding author upon reasonable request.

## References

[1] Wu J, Qui J, Khalil M, Ghaznavi S, Yeo C, Nohr E (2025). Prospective results for active surveillance of RAS-positive indeterminate thyroid nodules with Bethesda III and IV cytologies. Can J Diabetes.

[2] Sengul I, Sengul D (2021). Blurred lines for management of thyroid nodules in the era of atypia of undetermined significance/ follicular lesion of undetermined significance: novel subdivisions of categories IIIA and IIIB in a possible forthcoming The Bethesda System for Reporting Thyroid Cytopathology, 3rd edition; amending versus unnecessary?. Rev Assoc Med Bras (1992).

[3] Sengul I, Sengul D (2021). Focusing on thyroid nodules in suspense: 10-15 mm with repeat cytology, Category III, the Bethesda System for Reporting Thyroid Cytopathology, TBSRTC. Rev Assoc Med Bras (1992).

[4] Sengul D, Sengul I (2023). Subdivision of intermediate suspicion, the 2021 K-TIRADS, and category III, indeterminate cytology, the 2017 TBSRTC, 2nd edition, in thyroidology: let bygones be bygones?. Ultrasonography.

[5] Sengul I, Sengul D (2024). Evangely, the subcategorization has been announced in the 2023 Bethesda system for reporting thyroid cytopathology: let bygones be bygones in thyroidology!. Rev Assoc Med Bras (1992).

[6] Sengul I, Sengul D (2022). Comment on: "Evaluating treatment options in managing thyroid nodules with indeterminate cytology of TBSRTC in thyroidology: *addendum aut non*?". Rev Assoc Med Bras (1992).

[7] Sengul I, Sengul D (2022). May 25-31, International Thyroid Awareness Week & May 25, World Thyroid Day, 2022: indetermination of indeterminate cytology, AUS/FLUS, FN, SUSP, in Thyroidology?. Sanamed.

[8] Soares JM, Albayrak M, Sengul D, Sengul I (2024). Thyroid function after menopause: is there any concern in thyroidology?. Rev Assoc Med Bras (1992).

[9] Soares JM, Detanac D, Sengul I, Dugalic S, Sengul D, Detanac D (2024). Melatonin, menopause, and thyroid function in gynecologic endocrinology: what is the role?. Rev Assoc Med Bras (1992).

[10] Sengul D, Sengul I, Soares JM (2022). Repercussion of thyroid dysfunctions in thyroidology on the reproductive system: conditio sine qua non?. Rev Assoc Med Bras (1992).

